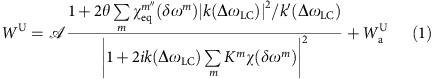# Erratum: Nonlinear detection of secondary isotopic chemical shifts in NMR through spin noise

**DOI:** 10.1038/ncomms15379

**Published:** 2017-04-19

**Authors:** Maria Theresia Pöschko, Victor V. Rodin, Judith Schlagnitweit, Norbert Müller, Hervé Desvaux

Nature Communications 8: Article number: 13914; DOI: 10.1038/ncomms13914 (2017); Published 01 09 2017; Updated 04 19 2017

This Article contains typographical errors in Equation 1, where the fraction line incorrectly extends across the entire equation. The correct form of Equation 1 is as follows: