# Supplementary material for: Nonlinear detection of secondary isotopic chemical shifts in NMR through spin noise
Source: Nat Commun. 2017 Jan 9;8:13914. doi: 10.1038/ncomms13914 (PMC5227550; doi:10.1038/ncomms13914)
Supplement: Supplementary Information — Supplementary Figures, Supplementary Table, Supplementary Methods and Supplementary References [file ncomms13914-s1.pdf]

SUPPLEMENTARY FIGURES

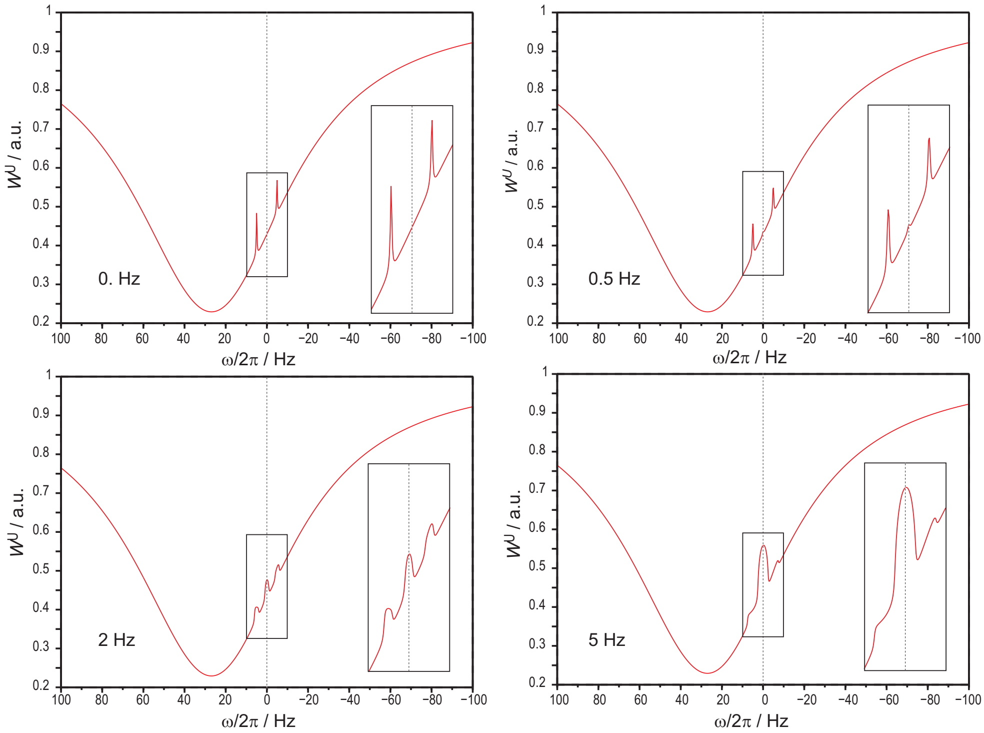

Figure 1. **Effect of the presence of a small static magnetic field gradient on a spin-noise spectrum.** The effect of small gradients consists in the appearance of a small bump at the exact resonance frequency of the main peak. For these numerical simulations, the sample was considered as a series of 101 slices with an applied magnetic field spreading the range of 0, 0.5 Hz, 2 Hz and 5 Hz, respectively. All other parameters are kept constant. The 0 Hz panel illustrates the reliability of the calculation, since, already for a 0.5 Hz line-broadening due to field inhomogeneity, a small bump is detectable. This bump is clearly visible for larger static magnetic field gradients. These bumps are always centered at the true chemical shift (0 Hz) used in the simulation. Numerically down to about 0.3 Hz, this bump starts to be visible on the raw data. But assessing this ability is strongly dependent on the resolution, the level of signal averaging and the spectrometer stability, since the “bump” should be distinguished from random fluctuations.

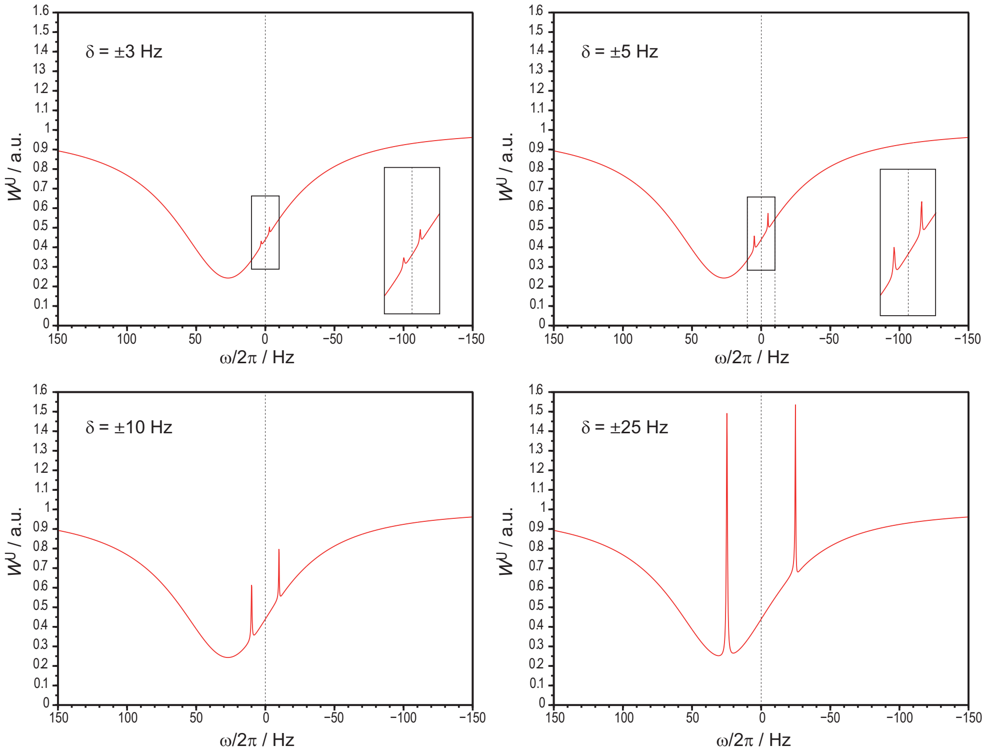

Figure 2. **Evaluation of the effect of chemical shift difference.** The high sensitivity of the enhancement on the offset δ is evident from the simulations of four different frequency offsets between the three species in a 100:1:1 concentration ratio (from $\delta = \pm 3$ to ± 25 Hz). Essentially when the frequency difference starts to be small when compared to the cross-precession terms,[1] they become indistinguishable from the main resonance. Finally if the main peak is strongly shifted by the frequency pushing contribution (its effective chemical shift is 0 Hz, even if the extremum of resonance line is at 27 Hz), the resonance frequencies of the minor ones are not shifted. The situation appears the more counterintuitive for the minor resonance whose chemical shift is $\delta = 25$ Hz, since the latter appears nicely as a narrow bump almost centered on the main large dip. This set of simulations illustrates that the difference in chemical shift δ between the strongly radiation-damping broadened peak and the minor component is more important than their exact values.

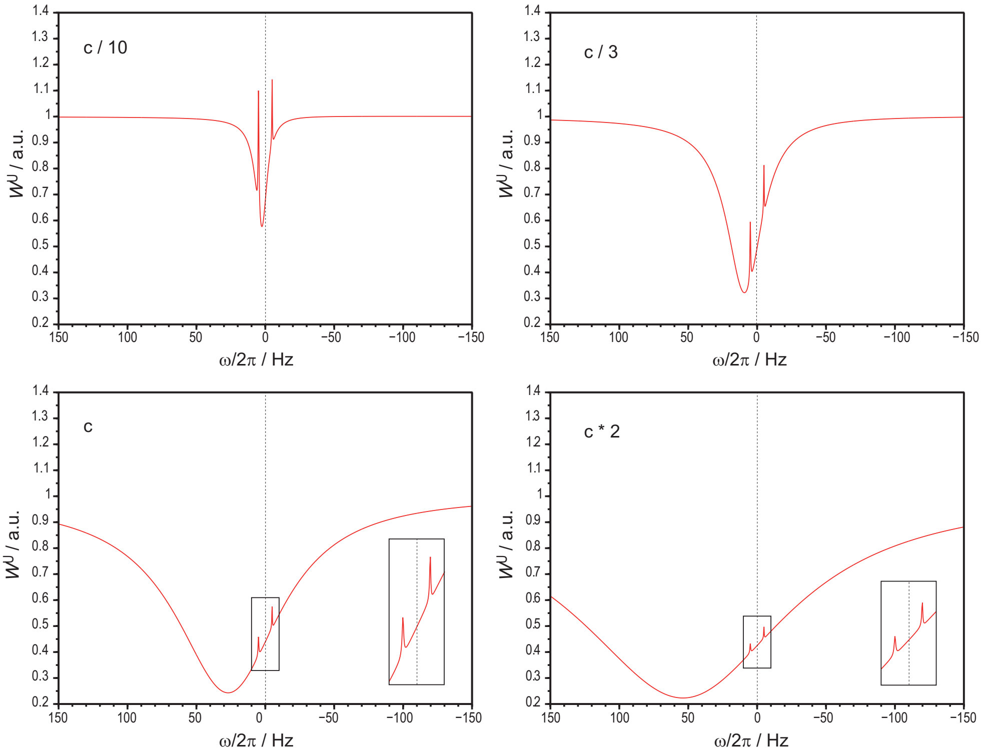

Figure 3. **Assessment of the concentration dependence.** Exploration of the influence of the radiation damping rate, λ_{r0}, on the detection capability of minor species is achieved by changing the concentration c of the main species, the minor ones where always 1/100 of the main one and $\delta = \pm 5\,\mathrm{Hz}$. As shown for room temperature probes[2], the radiation damping rate λ_{r0} can vary by more than one order of magnitude according to the transmission line length. Since on a commercial cooled-coil probe the amplitude of the changes is unknown also because the perfect tuning is expected to be very difficult to achieve[2], the dependence of the nuclear spin-noise spectral density on this parameter was explored by changing the spin concentration. Besides a more and more pronounced frequency pushing contribution experienced by the main resonance, the amplitudes of the peaks superimposing on the main dip are also strongly modified. For very large radiation damping (large concentration), the detection capability is reduced. Choosing very small concentrations is, in fact, precluded by the principle of noise measurements: the signal appears as a reduction or amplification of the average noise level. So the variation of noise signal amplitude due to the nuclear magnetization of the minor components should be compared to random fluctuations, amplitudes of which scale down as square root of signal averaging duration.

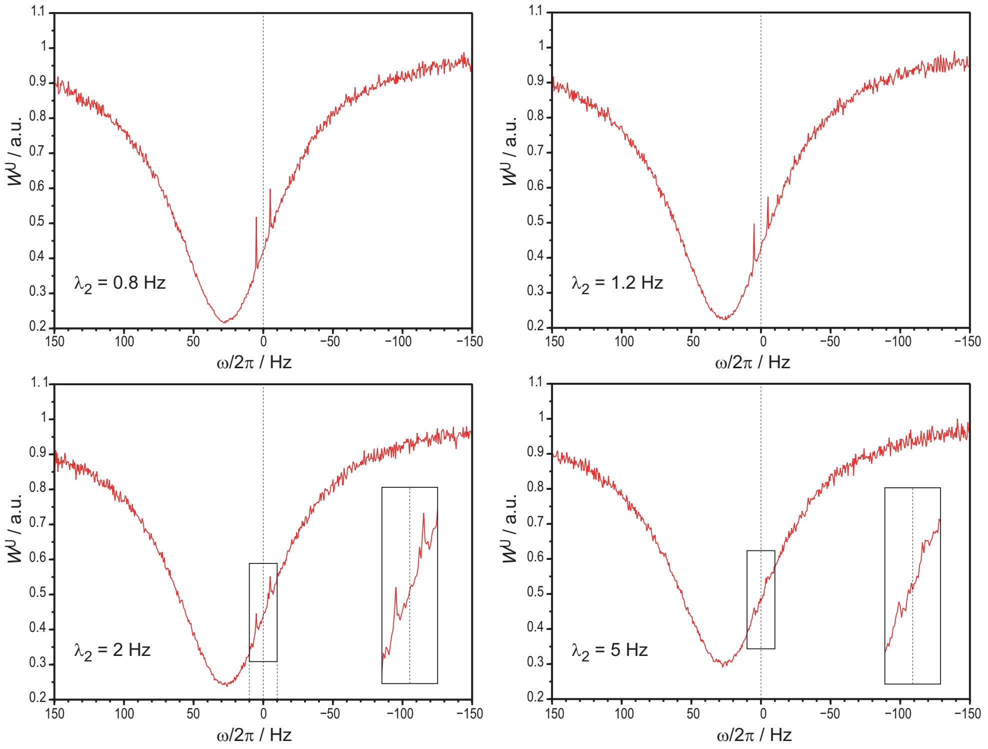

Figure 4. **Assessment of the influence of transverse relaxation.** The detection capability of secondary isotopic effects is dependent on the relative value of λ_2 and λ_r for a cooled-coil probe. Indeed their relative values induce a sign reversal of the signal from a bump to a dip[3]. The dependence is explored by varying the transverse relaxation rate values λ_2, all other parameters being kept constant. A spectral resolution of 0.5 Hz, signal averaging on 5 hours and sliding-windows processing approach[4,5] are assumed. For slow relaxing spins ($\lambda_2 = 0.8\,\text{Hz}$), strong narrow peaks are observed and in fact the limitations results from the digital resolution and the amount of signal averaging (time of signal averaging). For fast transverse relaxation ($\lambda_2 > 5\,\text{Hz}$), the bumps on the dip almost disappear and cannot be distinguished from random fluctuations.

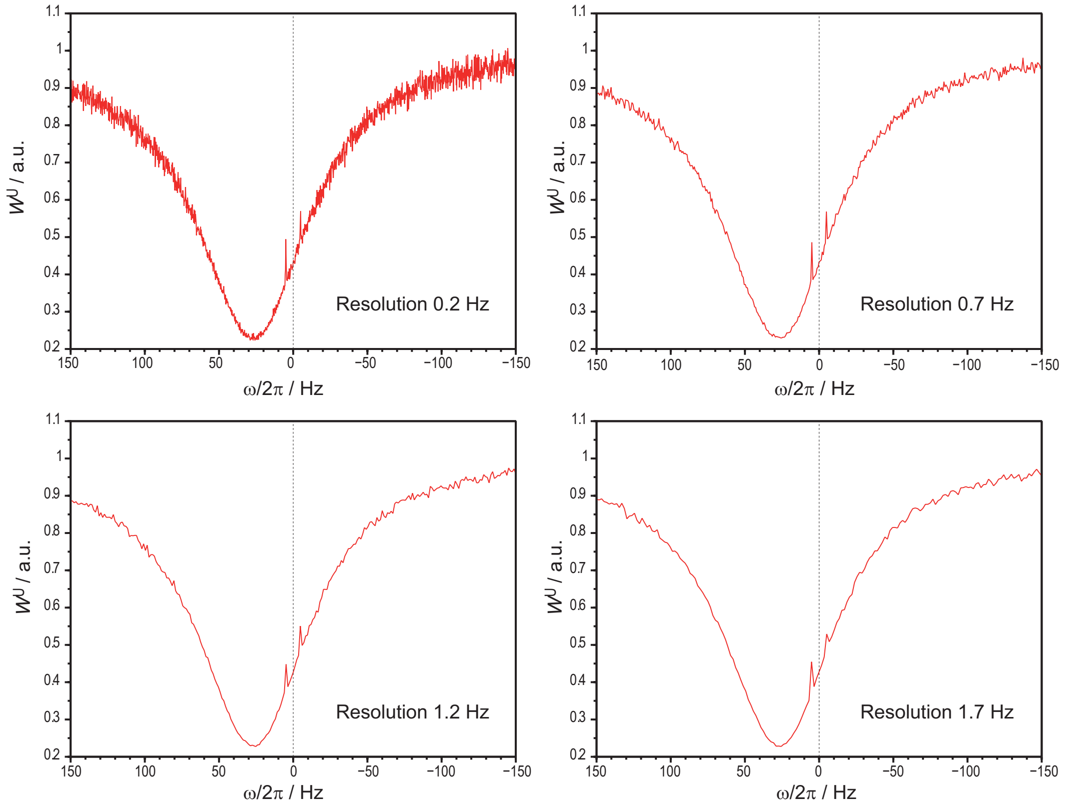

Figure 5. **Assessment of the digital resolution influence.** When detecting narrow bumps superimposed on a large dip, digitization and duration of time signal averaging affect the effective resolution of the signals and the detection capability. This is explored by keeping all parameters constant except the final resolution. A duration of 5 hours for signal averaging with the sliding-windows processing scheme is assumed[4,5]. This duration influences the amplitude of the fluctuations as a function of the spectral resolution. For very high resolutions, narrow bumps on the main dip can be detected, and they disappear for lower resolutions. Even if this dependence is significant, it is not an intrinsic limitation since with the sliding-window approach the final resolution is chosen during the processing. In fact, the achievable resolution depends on the long-term stability of the spectrometer. The latter can be assessed by comparing processed signals acquired at different moments, i.e. by comparing several spectra obtained from the same raw time-domain data, but by processing only part of them.

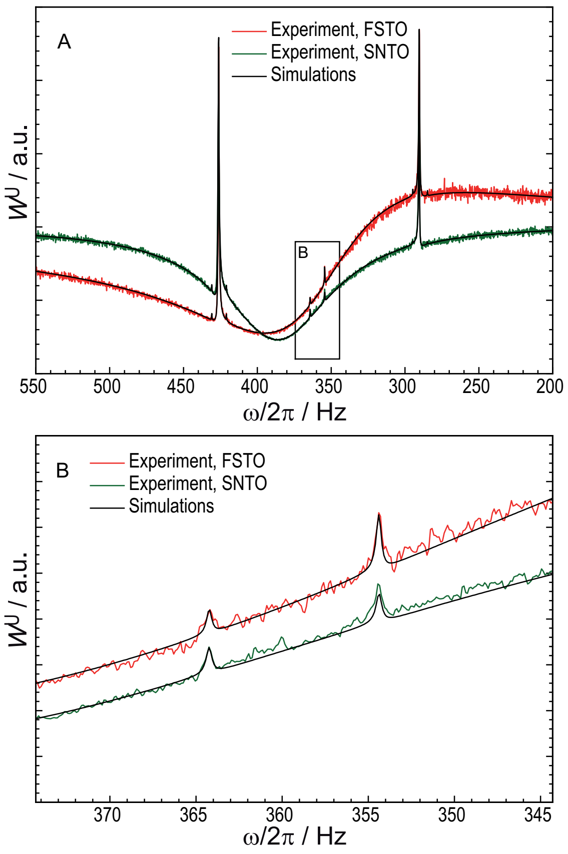

Figure 6. **Experimental and simulated spectra for two tuning conditions.** The green curve matches the one of Figure 3 in the main text and corresponds to the SNTO condition[3,6]. The red curve was obtained in the same way but at the FSTO condition[7]. The black curves are the best-fit theoretical curves to equation 5 of the main text. It allows a more accurate determination of the isotope-induced chemical shifts and coupling constants (parameters summarized in Table I). The secondary satellite signals were used for relative referencing of the spectra. Due to the nature of the non-linear fitting procedure and the absence of constraints on the electronic parameters, there is no guarantee that the set of deduced parameters, in particular for the electronic ones, is unique. The larger uncertainties are mainly in the electronic parameters and in the chemical shift of the main ($^{12}CH_3$) resonance. Indeed the position and "phase" of the main peak vary significantly due to its line-width and due to a significant frequency-shift contribution. The positions of the satellite peaks are largely independent of the tuning leading to very similar chemical shift and coupling constant values. As a consequence, in the absence of internal reference (for instance, a bump due to static magnetic field inhomogeneity) the accuracy of isotopic effect on chemical shifts appears limited by the accuracy on the determination of the absolute chemical shift of the main resonance.

SUPPLEMENTARY TABLE

Table I. Summary of parameters obtained by fitting Equation S50 to the experimental data.

	SNTO	FSTO
Amplitude	1.4307×10^9	6.7685×10^9
$\mathscr{A}$	0.30	0.50
$\mathscr{B}$	0.65	0.42
W_a^U	0.05	0.08
θ	15.5	15.5
λ_{r0}	108 Hz	123 Hz
$\arg(k(\Delta\omega_{LC}))$	$-29°$	$1°$
$\arg(\zeta)$	$-86°$	$-89°$
$\lvert\zeta\rvert$	0.74	1.01
$^{12}CH_3{-}^{12}CN$ (main resonance)		
$\omega_0/2\pi$	359.7 Hz	360.4 Hz
λ_2	1.3 Hz	1.3 Hz
$^{13}CH_3{-}^{12}CN$ (prim. satellite)		
$\omega_0/2\pi$	358.4 Hz	358.3 Hz
$^1J_{CH}$	136.2 Hz	136.2 Hz
λ_2	1.3 Hz	1.3 Hz
$^{12}CH_3{-}^{13}CN$ (sec. satellite)		
$\omega_0/2\pi$	359.3 Hz	359.3 Hz
$^2J_{CH}$	9.9 Hz	9.9 Hz
λ_2	1.3 Hz	1.1 Hz
$^{13}CH_3{-}^{13}CN$ (prim. and sec. satellite)		
$\omega_0/2\pi$	357.8 Hz	357.8 Hz
$^1J_{CH}$	136.2 Hz	136.2 Hz
$^2J_{CH}$	9.9 Hz	9.9 Hz
λ_2	1.0 Hz	1.0 Hz

SUPPLEMENTARY METHODS

A. Parameters for the simulated spectra

Nuclear spin-noise spectroscopy using cooled-coil probes relies on a non-linear response. As a consequence, detection capability, sensitivity, resolutions,... are strongly dependent on the experimental parameters. There are two types of parameters, those due to the electronic circuit ($\mathscr{A}, \mathscr{B}, k(\Delta\omega_{\mathrm{LC}}), \theta, \zeta, W_{\mathrm{a}}^{\mathrm{U}}$) and the spectroscopic ones ($\omega^m, \lambda_2^m, c^m, K^m$). Since in practice little is known on the former ones, in particular about their available range of relative variations due to severe constraints on the tuning conditions[2] but also on technical realization, only the dependence on the spectroscopic parameters were explored. The electronic parameters were chosen equal to the ones deduced from the best-fit theoretical curve to the experimental data (Figure 3 of the main text). For the simulations (Figures 1 to 5), the different electronic parameters were: $\mathscr{A} = 0.3$, $\mathscr{B} = 0.65$, $W_{\mathrm{a}}^{\mathrm{U}} = 0.05$, $\theta = 15.5$, $|\zeta| = 0.74$, $\arg(\zeta) = -86°$, $\arg(k(\Delta\omega_{\mathrm{LC}})) = -29°$, and the radiation damping rate for the concentration of the main peak $\lambda_{\mathrm{r0}} = 324\,\mathrm{Hz}$. The resonance frequency of this peak was used as a reference (0 Hz), the two satellites, concentrations of which were divided by 100 relative to the main peak, where shifted by $\pm\delta$ from the main one. λ_2 was chosen equal to 1.3 Hz.

B. Detailed description of the derivation of spin-noise spectral density

1. General concepts

In this subsection we shall consider that the detection circuit can be represented by a simple RLC circuit, with a coil of inductance L and resistance R and a capacitor C_{T} in parallel (Figure 4A of the main text). The measurement is done at the extremities of the capacitor chip (voltage U_2). There are two physical processes which contribute to the observation of nuclear spin-noise in NMR[8]:

- the fluctuations of the electric current in the coil induce transient $B_1(t)$ magnetic fields which may be able to excite, depending on their amplitudes and frequencies, the nuclear magnetization;
- the fluctuations of the transverse magnetization $\mathscr{M}_s(t)$ induce magnetic fields into the coil $-\imath\mu_0 k(\Delta\omega_{\mathrm{LC}})\mathscr{M}_s(t)$ which frequency is almost the spin Larmor resonance one, ω_0.

In these conditions, denoting μ_0 the magnetic permeability of free space, the time-dependent transverse magnetic field is:

$$B(t) = B_1(t) - \imath\mu_0 k(\Delta\omega_{\mathrm{LC}})[\mathscr{M}_s(t) + \mathscr{M}_r(t)] \tag{S1}$$

where the last source of rf field has been introduced. It corresponds to the feed-back field $-\imath\mu_0 k(\Delta\omega_{\mathrm{LC}})\mathscr{M}_r(t)$ induced by the precessing coherent transverse magnetization, $\mathscr{M}_r(t)$.

$k(\Delta\omega_{LC})$ is the coefficient of proportionality between the rf feed-back field and the precessing magnetization. It is dependent on the coil mistuning $\Delta\omega_{LC} = \omega_0 - \omega_{LC}$, with $\omega_{LC} = 1/\sqrt{LC_T}$ the circuit resonance frequency. One has[9]:

$$k(\Delta\omega_{LC}) = \frac{\eta Q\omega_0}{2\omega_{LC}} \frac{\exp(-\imath\psi)}{\sqrt{1+\Delta_{LC}^2}} \tag{S2}$$

with

$$|Z_c| = \frac{L\omega_{LC}}{Q}\sqrt{1+\Delta_{LC}^2} \tag{S3}$$

$$\Delta_{LC} = Q\frac{\omega_{LC}}{\omega}\left[\frac{\omega^2-\omega_{LC}^2}{\omega_{LC}^2}\right] \simeq 2Q\frac{\omega-\omega_{LC}}{\omega_{LC}} \tag{S4}$$

$$\psi = \arctan\Delta_{LC} \tag{S5}$$

where η is the coil filling factor and $Q = L\omega_{LC}/R$, the coil quality factor.

Following Sleator et al.[8], the spin dynamics can be described by considering the evolution of the transverse magnetization with the inclusion of these transient rf magnetic fields (equation S1). Considering a single-spin species, the equation would simply be:

$$\frac{d}{dt}\mathscr{M}_r + (\lambda_2 - \imath\omega_0)\mathscr{M}_r = -\imath\gamma\mathscr{M}_z B(t) \tag{S6}$$

Let us show that from these two equations (equations S1 and S6), the nuclear spin-noise spectra can be computed. The electric current inside the coil, $I(t)$, is proportional to the magnetic field $B(t)$:

$$I(t) = \sqrt{\frac{\mathscr{V}_s}{\mu_0 L\eta}}B(t) \tag{S7}$$

with $\mathscr{V}_s$ the sample volume. The electric current is proportional to the measured voltage U_2:

$$I(t) = jC_T\omega U_2(t) \tag{S8}$$

Thus the magnetic field is proportional to the monitored voltage:

$$U_2(t) = \alpha(\Delta\omega_{LC})B(t) \tag{S9}$$

with the coefficient $\alpha(\Delta\omega_{LC})$ which is dependent on the coil mistuning:

$$\alpha(\Delta\omega_{LC}) = \frac{1}{jC_T\omega}\sqrt{\frac{\mathscr{V}_s}{\mu_0 L\eta}} \tag{S10}$$

The nuclear spin-noise spectrum corresponds to the spectral density of the voltage fluctuations:

$$W^U = U_2(\omega)U_2^*(\omega) = |\alpha(\Delta\omega_{LC})|^2\overline{B(\omega)B^*(\omega)} \tag{S11}$$

Due to the dependence of $k(\Delta\omega_{LC})$ and $\alpha(\Delta\omega_{LC})$ on the tuning conditions, the calculation of the Fourier transform of equation S1 requires assumptions on the mistuning. The

separation between the frequency dependences of the nuclear spin and the electrical circuit is allowed thanks to their very different characteristic rates: $\omega_{\rm LC}/2Q$ for the electronic circuit ($> 10^5$ Hz) and the transverse relaxation rate λ_2 (~ 10 Hz) for the nuclear spins.

Equation S6 expressed in the frequency domain is:

$$-\imath\,\delta\omega\,\mathscr{M}_r(\delta\omega) + \lambda_2\mathscr{M}_r(\delta\omega) = -\imath\,\gamma\,\mathscr{M}_z B(\delta\omega) \tag{S12}$$

where the frequency difference, $\delta\omega = \omega_0 - \omega$, is considered in a range of values corresponding to the NMR spectrum close to spin Larmor frequency. In the following we shall consider cases where the magnetization is at equilibrium with $k_{\rm B}$ the Boltzmann constant and T the temperature:

$$\mathscr{M}_z = \mathscr{M}_0 = \frac{n\gamma\hbar^2\omega_0}{4k_{\rm B}T} \tag{S13}$$

Thus

$$\mu_0\mathscr{M}_r(\delta\omega) = 2\chi(\delta\omega)B(\delta\omega) \tag{S14}$$

where the complex nuclear magnetic susceptibility $\chi(\delta\omega)$ has been introduced:

$$\chi(\delta\omega) = \frac{\gamma\mu_0\mathscr{M}_z}{2}\frac{\delta\omega - \imath\lambda_2}{\lambda_2^2 + \delta\omega^2} \tag{S15}$$

In the frequency domain, equation S1 becomes:

$$\begin{aligned} B(\delta\omega) &= B_1(\delta\omega) - \imath\mu_0 k(\Delta\omega_{\rm LC})[\mathscr{M}_r(\delta\omega) + \mathscr{M}_s(\delta\omega)] \\ &= \frac{B_1(\delta\omega) - \imath\mu_0 k(\Delta\omega_{\rm LC})\mathscr{M}_s(\delta\omega)}{1 + 2\imath k(\Delta\omega_{\rm LC})\chi(\delta\omega)} \end{aligned} \tag{S16}$$

In equation S11, for the calculation of $\overline{B(\omega)B^*(\omega)}$ we can use the absence of correlations between fluctuations of $B_1(t)$ and $\mathscr{M}_s(t)$ since their physical origins (coil resistance and spins fluctuations, respectively) are different. Thus:

$$W^{\rm U} = |\alpha(\Delta\omega_{\rm LC})|^2\left[\frac{\overline{B_1(\omega)B_1^*(\omega)}}{|1 + 2\imath k(\Delta\omega_{\rm LC})\chi(\delta\omega)|^2} + \frac{\mu_0^2\overline{\mathscr{M}_s(\delta\omega)\mathscr{M}_s^*(\delta\omega)}|k(\Delta\omega_{\rm LC})|^2}{|1 + 2\imath k(\Delta\omega_{\rm LC})\chi(\delta\omega)|^2}\right] \tag{S17}$$

If we assume the electronic circuit to be perfectly tuned:

$$k(\Delta\omega_{\rm LC}) = k'(0) = \frac{\eta Q}{2} \tag{S18}$$

Introducing the radiation damping characteristic rate $\lambda_{\rm r}$:

$$\lambda_{\rm r} = \frac{\mu_0}{2}\eta Q\gamma\mathscr{M}_0 \tag{S19}$$

one finally obtains:

$$\begin{aligned} |1 + 2\imath k(0)\chi(\delta\omega)|^2 &= \left(1 + \frac{\lambda_2\lambda_{\rm r}}{\lambda_2^2 + \delta\omega^2}\right)^2 + \left(\frac{\delta\omega\lambda_{\rm r}}{\lambda_2^2 + \delta\omega^2}\right)^2 \\ &= \frac{\delta\omega^2 + (\lambda_2 + \lambda_{\rm r})^2}{\lambda_2^2 + \delta\omega^2} \end{aligned} \tag{S20}$$

In equation S17, following Sleator et al.[8], we have in the high temperature limit:

$$\overline{B_1(\omega)B_1^*(\omega)} = \frac{\mu_0}{4\pi}\frac{8\eta Q k_\mathrm{B} T}{\omega_0 \mathscr{V}_s}\frac{R^2}{|Z_\mathrm{c}|^2} \tag{S21}$$

and

$$\begin{aligned}\overline{\mathscr{M}_s(\delta\omega)\mathscr{M}_s^*(\delta\omega)} &= \frac{n\gamma^2\hbar^2}{\pi\mathscr{V}_s}\frac{\lambda_2}{\lambda_2^2+\delta\omega^2} \\ &= \frac{4k_\mathrm{B}T\gamma\mathscr{M}_0}{\pi\omega_0\mathscr{V}_s}\frac{\lambda_2}{\lambda_2^2+\delta\omega^2}\end{aligned} \tag{S22}$$

Finally by combining the different equations, and still considering perfect tuning conditions $\omega_\mathrm{LC} = \omega_0$, thus $Z_\mathrm{c} = R$:

$$W^\mathrm{U} = \frac{2Qk_\mathrm{B}T}{\pi C_\mathrm{T}\omega_0}\left[1 - \frac{\lambda_\mathrm{r}^2 + \lambda_\mathrm{r}\lambda_2}{\delta\omega^2 + (\lambda_2+\lambda_\mathrm{r})^2}\right] \tag{S23}$$

which corresponds to the nuclear spin-noise equation derived by McCoy and Ernst[10], when other noise sources such as that from the preamplifier, W_a^U, are added.

2. *Extension to a two-spin system*

We shall consider a two-spin system I^a and I^b and still the electronic circuit represented in Figure 4A of the main text. We label all parameters with a and b as superscripts, and introduce their Larmor frequencies ω^a and ω^b, respectively. We assume that there is no mechanism allowing an exchange of magnetization between these two species except the cross-precession ones[1]. Equations S1 and S6 can now be written for each species:

$$B(t) = B_1(t) - \imath\mu_0 k(\Delta\omega_\mathrm{LC})[\mathscr{M}_r^a(t) + \mathscr{M}_s^a(t) + \mathscr{M}_r^b(t) + \mathscr{M}_s^b(t)] \tag{S24}$$

$$\frac{d}{dt}\mathscr{M}_r^a + (\lambda_2^a - \imath\omega^a)\mathscr{M}_r^a = -\imath\gamma\mathscr{M}_z^a B(t) \tag{S25}$$

$$\frac{d}{dt}\mathscr{M}_r^b + (\lambda_2^b - \imath\omega^b)\mathscr{M}_r^b = -\imath\gamma\mathscr{M}_z^b B(t) \tag{S26}$$

In the frequency domain, the system becomes:

$$\mu_0\mathscr{M}_r^a(\delta\omega^a) = 2\chi^a(\delta\omega^a)B(\delta\omega) \tag{S27}$$

$$\mu_0\mathscr{M}_r^b(\delta\omega^b) = 2\chi^b(\delta\omega^b)B(\delta\omega) \tag{S28}$$

$$B(\delta\omega) = \frac{B_1(\delta\omega) - \imath\mu_0 k(\Delta\omega_\mathrm{LC})[\mathscr{M}_s^a(\delta\omega^a) + \mathscr{M}_s^b(\delta\omega^b)]}{1 + 2\imath k(\Delta\omega_\mathrm{LC})(\chi^a(\delta\omega^a) + \chi^b(\delta\omega^b))} \tag{S29}$$

Thus the expression for the noise spectral density becomes:

$$\begin{aligned}W^\mathrm{U} = |\alpha(\Delta\omega_\mathrm{LC})|^2 \Bigg[&\frac{\overline{B_1(\omega)B_1^*(\omega)}}{|1 + 2\imath k(\Delta\omega_\mathrm{LC})(\chi^a(\delta\omega^a) + \chi^b(\delta\omega^b))|^2} \\ + &\frac{\mu_0^2\overline{(\mathscr{M}_s^a(\delta\omega^a) + \mathscr{M}_s^b(\delta\omega^b))(\mathscr{M}_s^{a*}(\delta\omega^a) + \mathscr{M}_s^{b*}(\delta\omega^b))}|k(\Delta\omega_\mathrm{LC})|^2}{|1 + 2\imath k(\Delta\omega_\mathrm{LC})(\chi^a(\delta\omega^a) + \chi^b(\delta\omega^b))|^2}\Bigg]\end{aligned} \tag{S30}$$

where cross-terms between fluctuations of $\mathscr{M}_s^a$ and $\mathscr{M}_s^b$ are considered. For clarity reasons, we shall denote $\chi^a(\delta\omega^a) + \chi^b(\delta\omega^b) = \chi$ and similarly for $\mathscr{M}_{r,s} = \mathscr{M}_{r,s}^a + \mathscr{M}_{r,s}^b$. In equation S30, we have the spectral density of the $B_1(\delta\omega)$ field which was defined in Equation S21 and the spectral density of the sum of the fluctuating magnetization. For computing the latter, we can follow the strategy used by Sleator et al.[8] and compute the power flow from the spins into the circuit ΔP, averaged over a time $T_0 \longrightarrow \infty$:

$$\Delta P = \lim_{T_0 \longrightarrow \infty} \int_{-\infty}^{\infty} -\mathscr{V}_s \omega_0 \mathrm{Im}\Big([\mathscr{M}_r^*(\delta\omega) + \mathscr{M}_s^*(\delta\omega)] B(\delta\omega) \Big) d(\delta\omega) \tag{S31}$$

Since $\mu_0 \mathscr{M}_r(\delta\omega) = 2\chi B(\delta\omega)$ with $B(\delta\omega)$ defined in equation S29, one obtains (the explicit $\delta\omega$ dependencies are dropped for clarity):

$$\begin{aligned} \Delta P &= \lim_{T_0 \longrightarrow \infty} \int_{-\infty}^{\infty} -\mathscr{V}_s \omega_0 \left(\mathrm{Im}(2\mu_0 \chi^* |B|^2) + \mathrm{Im}(\mathscr{M}_s^* B) \right) d(\delta\omega) \\ &= \lim_{T_0 \longrightarrow \infty} \int_{-\infty}^{\infty} -\mathscr{V}_s \omega_0 \left(-2\mu_0 \chi'' |B|^2 - \mu_0 \mathrm{Im}(\mathscr{M}_s^* (\imath k)(\mathscr{M}_r + \mathscr{M}_s)) \right) d(\delta\omega) \end{aligned} \tag{S32}$$

where we used the fact that there is no correlation between fluctuations of $\mathscr{M}_s$ and B_1. One has:

$$\begin{aligned} |B|^2 &= \left| B_1 - \imath k \frac{2\chi B_1 - \imath 2k\chi\mu_0 \mathscr{M}_s}{1 + 2\imath k\chi} - \imath \mu_0 k \mathscr{M}_s \right|^2 \\ &= \frac{|B_1|^2}{|1 + 2\imath k\chi|^2} + \frac{\mu_0^2 |k\mathscr{M}_s|^2}{|1 + 2\imath k\chi|^2} \end{aligned} \tag{S33}$$

and

$$\begin{aligned} \mu_0 \mathrm{Im}(\mathscr{M}_s^* (\imath k)(\mathscr{M}_r + \mathscr{M}_s)) &= -\mu_0 |\mathscr{M}_s|^2 \mathrm{Im}(\imath k) + |\mathscr{M}_s|^2 \mathrm{Im} \frac{2\mu_0 \chi k^2}{1 + 2\imath \chi k} \\ &= -|\mathscr{M}_s|^2 \mathrm{Im}\left(\imath \mu_0 k + \frac{2\mu_0 k^2 \chi^*}{1 + 2\imath k\chi} \right) \end{aligned} \tag{S34}$$

At the end:

$$\Delta P = \lim_{T_0 \longrightarrow \infty} \int_{-\infty}^{\infty} \mathscr{V}_s \mu_0 \omega_0 \frac{-2\chi'' |B_1(\delta\omega)|^2 + k' |\mathscr{M}_s(\delta\omega)|^2}{|1 + 2\imath k\chi|^2} d(\delta\omega) \tag{S35}$$

The spectral density of $\mathscr{M}_s$ can be deduced from the absence of power flow when the probe and the spins are at the same temperature. So:

$$\overline{|\mathscr{M}_s(\delta\omega)|^2} = \frac{2\chi''}{k'} \overline{|B_1(\delta\omega)|^2} \tag{S36}$$

which is a relation in agreement with the fluctuation-dissipation theorem[11,12].

Equation S30 becomes:

$$W^{\mathrm{U}} = \overline{|B_1(\delta\omega)|^2}\, |\alpha(\Delta\omega_{\mathrm{LC}})|^2 \frac{1 + 2\chi'' |k(\Delta\omega_{\mathrm{LC}})|^2 / k'(\Delta\omega_{\mathrm{LC}})}{|1 + 2\imath k(\Delta\omega_{\mathrm{LC}})\chi|^2} \tag{S37}$$

We consequently deduce for a perfectly tuned probe $\omega_{\rm LC} = \omega_0$ and after reintroducing the whole expressions for the different susceptibilities:

$$W^{\rm U} = \frac{2Qk_{\rm B}T}{\pi C_{\rm T}\omega_0} \frac{1 + \frac{\lambda_{\rm r}^a\lambda_2^a}{\lambda_2^{a2}+\delta\omega^{a2}} + \frac{\lambda_{\rm r}^b\lambda_2^b}{\lambda_2^{b2}+\delta\omega^{b2}}}{\left(1 + \frac{\lambda_{\rm r}^a\lambda_2^a}{\lambda_2^{a2}+\delta\omega^{a2}} + \frac{\lambda_{\rm r}^b\lambda_2^b}{\lambda_2^{b2}+\delta\omega^{b2}}\right)^2 + \left(\frac{\lambda_{\rm r}^a\delta\omega^a}{\lambda_2^{a2}+\delta\omega^{a2}} + \frac{\lambda_{\rm r}^b\delta\omega^b}{\lambda_2^{b2}+\delta\omega^{b2}}\right)^2} \tag{S38}$$

3. Generalization to n-spin systems, cooled-coil probe and hyperpolarized systems

Equation S37 is valid for a two-spin system with $\chi = \chi^a(\delta\omega^a) + \chi^b(\delta\omega_b)$. The case of n spins is directly obtained by redefining χ as the sum of all susceptibilities. The case of hyperpolarized species or cryo-probe are considered by replacing in equation S37:

- the χ'' in the numerator by its value at thermal equilibrium $\chi''_{\rm eq}$, since it results from the fluctuations of the transverse magnetization which is independent of the nuclear polarization but only depends on the number of spins[10];
- multiplying this term by the sample-to-coil temperature ratio θ, since the coefficient $|B_1(\delta\omega)|^2$ is scaled down by the low temperature of the coil[3];
- finally in the denominator the susceptibility has to take into account the enhancement factor (K^m) of the $m^{\rm th}$ spin species[4]; this enhancement could be due to hyperpolarization or saturation ($K^m = 0$); at thermal equilibrium $K^m = 1$.

Finally by considering a constant preamplifier noise $W^{\rm U}_{\rm a}$ as historically done[10], the observed spectral densities can be represented by (also equation 1 of the main text):

$$W^{\rm U} = \mathscr{A}\frac{1 + 2\theta\sum_m \chi^{m\prime\prime}_{\rm eq}(\delta\omega^m)|k(\Delta\omega_{\rm LC})|^2/k'(\Delta\omega_{\rm LC})}{|1 + 2\imath k(\Delta\omega_{\rm LC})\sum_m K^m\chi(\delta\omega^m)|^2} + W^{\rm U}_{\rm a} \tag{S39}$$

where $\mathscr{A}$ is the noise contribution of the coil.

4. Extension to the case of finite input impedance of the preamplifier

The last aspect which has to be included in the description of the nuclear spin-noise spectra in NMR concerns the influence of the preamplifier noise and of the transmission line which tends to induce that the Spin-Noise Tuning Optimum (pure in-phase Lorentzian shape for nuclear resonances) can be different to Frequency-Shift Tuning Optimum (vanishing of the frequency-pushing contribution)[7]. Therefore, one should consider the electronic circuit of Figure 4B of the main text for which the input impedance of the preamplifier Z is not infinite.

The electronic model of Figure 4B of the main text corresponds to the addition of a new noise source: the Johnson-Nyquist noise of the internal resistance of the preamplifier (V_p).

The associated fluctuations induce transient electric currents in the coil and thus transient rf fields which are able to excite the nuclear magnetization. Due to impedance mismatches and the presence of the transmission line of impedance Z_0, the resonance frequencies of the electronic circuit seen from the coil or from the preamplifier are usually different[2]. Starting from this principle, the derivation of an analytical or a numerical model is inhibited by the large number of unknown electronic elements present in the circuit and of their values. Nevertheless, since our aim resides in obtaining an analytical equation for describing the nuclear spin-noise spectra, instead of considering all electronic components, we shall consider a transmission matrix T

$$T = \begin{pmatrix} T_{11} & T_{12} \\ T_{21} & T_{22} \end{pmatrix}$$

which connects the voltages and intensities at the input impedance of the preamplifier (U_1, I_1) and at the extremities of the coil (U_2, I_2). With the sign convention of Figure 4B of the main text, we have the following relations:

$$\begin{pmatrix} U_2 \\ I_2 \end{pmatrix} = \begin{pmatrix} T_{11} & T_{12} \\ T_{21} & T_{22} \end{pmatrix} \begin{pmatrix} U_1 \\ I_1 \end{pmatrix}; \qquad \begin{pmatrix} U_1 \\ I_1 \end{pmatrix} = \begin{pmatrix} T_{22} & -T_{12} \\ -T_{21} & T_{11} \end{pmatrix} \begin{pmatrix} U_2 \\ I_2 \end{pmatrix} \tag{S40}$$

The different noise sources (resistance of the preamplifier, nuclear magnetization and resistance of the coil) being uncorrelated, the solution can be obtained by successively considering them and applying the principle of superposition for computing the voltage $U_z = U_z^c + U_z^p$ and spectral density W^{U} where U_z^c is the measured voltage due to fluctuations occurring in the coil (magnetization and coil resistance) and U_z^p to that due to the preamplifier impedance.

We shall start by considering the electronic circuit of Figure 4B of the main text and only the fluctuations due to the coil resistance and due to the magnetization (voltage sources: $V \neq 0$ and $V_p = 0$). By considering the equivalent impedance Z_{eq} at the plane number ② due to the left part of the electronic circuit, in a range of frequencies close to resonance, equation S2 is still valid after redefining the different parameters[2,9]. Moreover there is still a proportionality coefficient $\alpha(\Delta\omega_{\mathrm{LC}})$ between the measured voltage and the rf field. Indeed, the measured voltage $U_z^c = U_1$ verifies:

$$U_z^c = T_{22} U_2 - T_{12} I_2 \tag{S41}$$

and the voltage and intensity at the coil extremities verify:

$$U_2 = V + (jL\omega + R) I_2 = -Z_{\mathrm{eq}} I_2 \tag{S42}$$

where V is the voltage created by the precessing magnetization[9], the magnetization fluctuations and by the Johnson-Nyquist noise of the coil resistance V_c.

$$V = \sqrt{\mu_0 \eta L}\,\omega(\mathscr{M}_r + \mathscr{M}_s) + V_c \tag{S43}$$

where we have neglected the difference of resonance frequencies between the different spins. By combining equations S41 and S42, the measured voltage U_z^c is found proportional to V:

$$U_z^c = \frac{Z_{\text{eq}} T_{22} + T_{12}}{Z_{\text{eq}} + jL\omega + R} V \tag{S44}$$

Finally using equation S42, the voltage V is found proportional to the electric current inside the coil I_2, that is to the magnetic field B (equation S7). Thus the measured voltage U_z^c is proportional to the magnetic field B. The transformation of a simple RLC circuit to the more complex circuit of Figure 4B of the main text only modifies the proportionality coefficient $\mathscr{A}$ but not the frequency dependence of equation S39 for the nuclear spin noise. This derivation is, in fact, an extension to the n-spin system case of that presented in Section III.B of Reference 2.

In the second step, we consider now as noise source V_p, only resulting from the preamplifier real impedance, and derive the induced value of the measured voltage U_z^p. As a consequence, the voltage V is now only due to the precessing magnetization, $\mathscr{M}_r$. Using the transmission matrix T, we have the following relations:

$$\begin{aligned} U_z^p &= U_1 - V_p = T_{22} U_2 - T_{12} I_2 - V_p \\ &= T_{22}\Big(V + (jL\omega + R) I_2\Big) - T_{12} I_2 - V_p \\ &= -Z I_1 = Z T_{21}\big(V + (jL\omega + R) I_2\big) - Z T_{11} I_2 \end{aligned} \tag{S45}$$

The electric current I_2 is consequently equal to:

$$I_2 = \frac{(Z T_{21} - T_{22}) V + V_p}{(Z T_{11} - T_{12}) + (jL\omega + R)(T_{22} - Z T_{21})} \tag{S46}$$

Introducing I_2 of equation S46 into equation S45, the expression of U_z functions of V and V_p is derived. Let us write it as a linear combination:

$$U_z^p = aV + bV_p \tag{S47}$$

where a and b are two complex-number parameters dependent on the electronic components. a and b have a negligible frequency dependence within the NMR-spectrum frequency range, since they only depend on the electronic elements.

Using the previously derived relations between the transverse magnetization $\mathscr{M}_r$ and the RF field which excites the magnetization, the voltage V produced by the precessing magnetization can be written as:

$$\begin{aligned} V &= \sqrt{\mu_0 \eta L}\,\omega \mathscr{M}_r \\ &= \sqrt{\frac{\eta L}{\mu_0}}\,\omega \frac{2 B_1^p(t) \sum_m K^m \chi^m(\delta\omega^m)}{1 + 2\imath k(\Delta\omega_{\text{LC}}) \sum_m K^m \chi^m(\delta\omega^m)} \end{aligned} \tag{S48}$$

Moreover, the fluctuations of the RF field $B_1^p(t)$ induced by the voltage V_p in the absence of magnetization (thus $V = 0$) can be obtained:

$$\begin{aligned} U_2 &= T_{11}(V_p + Z I_1) + T_{12} I_1 = (jL\omega + R) I_2 \\ I_1 &= T_{11} I_2 - T_{21} U_2 = T_{11} I_2 - T_{21}(jL\omega + R) I_2 \end{aligned}$$

By combining these two equations, V_p is found proportional (complex-number) to the electric current inside the coil I_2. Thus the RF fluctuations $B_1^p(t)$ are proportional to V_p. V and V_p are consequently proportional to the fluctuating magnetic field $B_1^p(t)$.

Finally the spectral density due to the preamplifier noise $W_p = |U_z^p|^2$ can be written as:

$$W_p = \mathscr{B} \frac{|1 + 2\imath\zeta \sum_m K^m \chi^m(\delta\omega^m)|^2}{|1 + 2\imath k(\Delta\omega_{\mathrm{LC}}) \sum_m K^m \chi^m(\delta\omega^m)|^2} \tag{S49}$$

where $\mathscr{B}$ is a real positive value and ζ a complex-number parameter dependent on the electronic circuit parameters.

The overall noise spectral density is obtained by the summation of the different contributions (equation 5 of the main text):

$$\begin{aligned} W = \mathscr{A} & \frac{1 + 2\theta \sum_m \chi_{\mathrm{eq}}^{m\,\prime\prime}(\delta\omega^m) |k(\Delta\omega_{\mathrm{LC}})|^2 / k'(\Delta\omega_{\mathrm{LC}})}{|1 + 2\imath k(\Delta\omega_{\mathrm{LC}}) \sum_m K^m \chi^m(\delta\omega^m)|^2} + \\ & + \mathscr{B} \frac{|1 + 2\zeta\imath k(\Delta\omega_{\mathrm{LC}}) \sum_m K^m \chi^m(\delta\omega^m)|^2}{|1 + 2\imath k(\Delta\omega_{\mathrm{LC}}) \sum_m K^m \chi^m(\delta\omega^m)|^2} + W_{\mathrm{a}}^{\mathrm{U}} \end{aligned} \tag{S50}$$

SUPPLEMENTARY REFERENCES

[1]Schlagnitweit, J., Morgan, S. W., Nausner, M., Müller, N. & Desvaux, H. Non-linear signal detection improvement by radiation damping in single-pulse NMR spectra. *ChemPhysChem* **13,** 482-487. (2012).

[2]Ferrand, G., Huber, G., Luong, M. & Desvaux, H. Nuclear spin noise in NMR revisited. *J. Chem. Phys.* **143,** 094201. (2015).

[3]Nausner, M., Schlagnitweit, J., Smrecki, V., Yang, X., Jerschow, A. & Müller, N. Non-linearity and frequency shifts of nuclear magnetic spin-noise. *J. Magn. Reson.* **198,** 73-79. (2009).

[4]Desvaux, H., Marion, D. J. Y., Huber, G. & Berthault, P. Nuclear spin-noise spectra of hyperpolarized systems. *Angew. Chem. Int. Ed.* **48,** 4341-4343. (2009).

[5]Desvaux, H. Non linear liquid-state NMR. *Prog. NMR Spectrosc.* **70,** 50-71, doi:10.1016/j.pnmrs.2012.11.001. (2013).

[6]Marion, D. J. Y. & Desvaux, H. An alternative tuning approach to enhance NMR signal. *J. Magn. Reson.* **193,** 153-157. (2008).

[7]Pöschko, M. T., Schlagnitweit, J., Huber, G., Nausner, M., Horničáková, M., Desvaux, H. & Müller, N. On the tuning of high resolution NMR probes. *ChemPhysChem* **15,** 3639-3645. (2014).

[8]Sleator, T., Hahn, E. L., Hilbert, C. & Clarke, J. Nuclear-spin noise and spontaneous emission. *Phys. Rev. B* **36,** 1969-1980. (1987).

[9]Vlassenbroek, A., Jeener, J. & Broekaert, P. Radiation damping in high resolution liquid NMR: a simulation study. *J. Chem. Phys.* **103,** 5886-5897. (1995).

[10]McCoy, M. A. & Ernst, R. R. Nuclear spin noise at room temperature. *Chem. Phys. Lett.* **159,** 587-593. (1989).

[11]Callen, H. B. & Welton, T. A. Irreversibility and generalized noise. *Phys. Rev.* **83,** 34-40 (1951).

[12]Marconi, U. M. B., Puglisi, A., Rondoni, L. & Vulpiani, A. Fluctuation-dissipation: Response theory in statistical physics. *Phys. Rep.-Rev. Sec. Phys. Lett.* **461,** 111-195 (2008).
